# DBOS: A Dialog-Based Object Query System for Hospital Nurses

**DOI:** 10.3390/s20226639

**Published:** 2020-11-19

**Authors:** Edward T.-H. Chu, Zi-Zhe Huang

**Affiliations:** Department of Computer Science and Information Engineering, National Yunlin University of Science and Technology, Yunlin 64002, Taiwan; kikororo7277@gmail.com

**Keywords:** natural language processing, conversational agent, instant messenger, smart hospital

## Abstract

Due to the advance of indoor positioning technology, it is now possible to trace mobile medical equipment (such as electrocardiography machines, patient monitors, and so on) being moved around a hospital ward. With the support of an object tracking system, nurses can easily locate and find a device, especially when they prepare for a shift change or a medical treatment. As nurses usually face high workloads, it is highly desirable to provide nurses with a user-friendly search interface integrated into a popular mobile app that they use daily. For this, DBOS, a dialog-based object query system, is proposed, which simulates a real conversation with users via the Line messaging app’s chatbot interface. A hybrid method that combines cosine similarity (CS) and term frequency–inverse document frequency (TF-IDF) is used to determine user intent. The result is returned to the user through Line’s interface. To evaluate the applicability of DBOS, 70 search queries given by a head nurse were tested. DBOS was compared with CS, TF-IDF, and Facebook Wit.ai respectively. The experiment results show that DBOS outperforms the abovementioned methods and can achieve a 92.8% accuracy in identifying user intent.

## 1. Introduction

With the advance of IoT (Internet of Things) and indoor positioning technology, object tracking applications has attracted more attention in hospitals recently. For example, in a hospital ward, mobile medical equipment, such as electrocardiography machines, patient monitors, and so on, is usually shared by rooms and could be scattered around the ward. As a standard operating procedure, nurses need to make notes on paper or a whiteboard whenever a medical device is moved to a different room or a different place. However, manually recording the location of a medical device is inefficient and may cause human error, especially when nurses work under high pressure. Once there is an error in the notes, it becomes time consuming to search for a medical device room by room when nurses prepare for a shift change or a medical treatment. With the support of an indoor object tracking system, nurses can easily locate and search for a device. However, nurses usually face high workloads and may be too busy to type search commands when walking around a ward. Sometimes, they try to avoid touching the keyboard and mouse when wearing gloves during their daily work [1,2]. Providing them with a user-friendly search interface integrated into a popular mobile app that they use daily is highly desirable. Therefore, the goal of this work is to design a flexible and effective dialog-based object query system for hospital nurses.

A dialog-based object query system needs a natural language processing (NLP) engine to understand users’ query intent before it can return a correct search result. Generally speaking, NLP is classified into three categories: rule-based, machine-learning-based, and feature-based. R. P. Mahapatra et al. [3] used AIML (artificial intelligence markup language) to match user input strings with a rule knowledge base, where hundreds and thousands of rules are stored. The accuracy of intent classification highly depends on the size of the rule knowledge base. The more rules the knowledge base has, the more accurate it will be. However, enumerating all possible rules is time consuming and impractical in real-world applications. S. Jaf et al. [4] first adopted a parse tree to describe the structure of user’s sentences. They then used bidirectional long short-term memory (BiLSTM), an artificial recurrent neural network (RNN), to overcome the ambiguity in natural language sentences. However, to have a good classification result, large volumes of training data may be required. For feature-based NLP methods, P. P. Gokul et al. [5] used cosine similarity (CS) to calculate the similarity between the user input string and annotated strings. Without differentiating the importance of each word in a sentence, the proposed method may fail to identify user intent from the cosine similarity. The situation becomes worse especially when there are many ignorable words in a sentence, such as “the”, “is”, “at”, and so on.

In this paper, DBOS, i.e., dialog-based object query system, is proposed, which simulates a real conversation with users via the Line messaging app’s chatbot interface [6]. DBOS is an extension of our previous work SBOT (social media-based object tracking system) [7], which combines the popular social media app Line with an indoor object tracking system. Inside DBOS, there is an intent table, stored at a remote server, that lists possible query intents in a nurse’s daily working scenario, such as search for a device, search for all devices, search for inventory, search for available devices, and so on. After receiving a user’s input, DBOS first performs synonym expansion to avoid possible misjudgment. Then, a hybrid method that combines cosine similarity (CS) and term frequency–inverse document frequency (TF-IDF) is used to determine user intent. Finally, DBOS returns the location of found objects to the user through Line’s interface. In the implementation, Jieba [8], a Chinese word segmentation tool, is adopted for preprocessing, which includes sentence segmentation, lowercase conversion, empty spaces removal, and marks removal. When integrating TF-IDF with CS, proper weights are assigned to different words in TF-IDF to avoid possible ambiguity in user intent. In order to evaluate the applicability of DBOS, 70 search queries given by a head nurse are tested, and DBOS is compared with CS, TF-IDF, and Facebook Wit.ai. The experiment results show that the accuracy rate of DBOS in identifying user intent is 92.8%, while that of CS is 70%, TF-IDF is 74.2%, and Facebook Wit.ai is 72.8%.

The rest of this paper is organized as follows. Section 2 surveys related works in NLP and discusses their limitations. Section 3 presents the design requirements and the method of DBOS. Finally, the experiment results and conclusion are given in Section 4 and Section 5 respectively.

## 2. Related Work

Many research efforts have been made for the development of NLP methods. They are classified into three categories: rule-based methods, machine-learning-based methods, and feature-based methods. In addition, several projects were led by technology giants to provide research communities and developers with NLP platforms and tools. This section discusses the representative work in the three categories followed by several well-known NLP platforms.

### 2.1. Rule-Based Methods

Rule-based methods use keywords to form rules to detect a user’s intent. If the user’s input matches a rule, the user’s intent is determined. On the contrary, when the input cannot match any predefined rules, the user’s intent is undetermined. G. Saqib et al. [9] used the ready-made AIML (artificial intelligence markup language) rule knowledge base to classify user input strings. Hundreds and thousands of rules were predefined. W. Y. Gang et al. [10] proposed a Chinese university consulting system based on AIML. However, if there is an error or ambiguity in word segmentation, synonyms expansion, or word ordering, it becomes difficult to have a rule-based classification system with an appropriate number of rules. R. Pirrone et al. [11] further combined AIML with graphics to dynamically generate a dialog box for users to choose answers from. According to the investigation conducted by Z. Zeng et al. [12], constructing a knowledge base for a professional field requires a huge amount of manual work, even for professionals. N. T. Thomas [13] proposed an e-commerce chat system that used latent semantic analysis together with AIML rule knowledge base to identify a user’s question. In summary, the accuracy of rule-based NLP methods highly depends on the scale and completeness of the rule knowledge base. If a user query can be expressed in multiple ways, all possible rules should be included. However, enumerating all possible rules is time consuming and impractical for many real-world applications. Scalability is a common problem of the rule-based classification.

### 2.2. Machine-Learning-Based Methods

Machine-learning-based NLP usually includes expression segmentation, vector conversion, and neural network training. J. Gu et al. [14] proposed an utterance-to-utterance interactive matching network (U2U-IMN) to calculate the relationship between the utterances in the context. If the context is short, the proposed work may not be able to recognize the input. Similarly, R. Singh et al. [15] proposed an automated customer service chatbot that can respond to users’ queries. However, when the proposed method converts training data into vectors, sentences are converted into the bag-of-words model. This will cause data sparseness issues [16] and will therefore reduce the accuracy, especially when the features of the training data are insufficient. B. Liu et al. [17] proposed a deep neural network (DNN) model that can have a proper reaction according to users’ personalities. However, additional training data are required if input messages are short. Their method is not suitable for the current application because search queries issued by nurses are usually short. H. Yutai et al. [18] proposed a sequence-to-sequence generation-based data augmentation framework that utilizes one utterance’s same semantic alternatives in the training data. Short training sentences were included. J. Li et al. [19] proposed a bidirectional long short-term memory (Bi-LSTM) network architecture for text classification. The proposed system conducted one-hot encoding on text data when it performed preprocessing. However, when the proposed method is applied to an application with numerous classification categories, such as NLP, the one-hot encoding will induce many problems. Examples include a large feature dimension, curse of dimensionality, high complexity, and long training time [20,21]. In summary, a language can be regarded as a discrete symbol system. Each character or word is a discrete random variable. Therefore, machine-learning-based methods often encounter data sparseness problems and curse of dimensionality. To address these problems, high-quality training data are required. In most cases, a large amount of training data are also required to have a good classification result. We admit that a machine-learning-based method could be a solution for our case; however, the computation complexity of a machine-learning method is usually high and may not be affordable for every hospital, especially when there is a need for hospital-wide deployment. Therefore, machine-learning technology was not adopted in this work.

### 2.3. Feature-Based Methods

Methods that used statistical text features are regarded as feature-based NLP methods. L. Hidayatin et al. [22] used cosine similarity (CS) to calculate the text similarity between the user input string and the questions in a database. Similarly, S. A. Abdul-Kader et al. [23] used Jaccard’s coefficient to calculate the union ratio of strings. Without differentiating the importance of each word in a sentence, their methods may fail to identify user intent. B. Su et al. [24] proposed an improved TF-IDF method that used the number of Google search results as the weight of TF. In their method, the number of pages was inversely proportional to the importance of the word. However, this method has a significant deviation in judging synonyms. Similarly, Z. Zhu et al. [25] proposed a refined term frequency inversed document frequency algorithm, called TA TF-IDF, to increase the IDF weight of importance words based on the word’s news exposure rate. However, the performance highly depends on the quality of selected news. In additional, many professional terminologies are rarely shown in news, which can limit the applicability of their method. J. Niu et al. [26] used BM25 to weight and rank short texts. Similar to TF-IDF, BM25 uses term frequency and IDF as the ranking basis. The difference is that BM25 uses free parameters to limit the maximum value of TF and adjust the effect of text length on the similarity score. However, there is no systematic way to find proper parameter values. A. I. Kadhim et al. [27] compared the feature extraction performance of BM25 and TF-IDF. The results showed that TF-IDF performed slightly better than BM25. The major reason is that TF-IDF uses two different tools, i.e., local formula TF and the global formula IDF, for classification. In addition, BM25 sometimes got an opposite effect on performance when it limits high-frequency words. In short, the major advantage of a feature-based method is that it directly calculates the similarity of texts based on the term frequency, word importance, and rarity of the text. Feature-based methods do not require a lot of training data. However, considering various nonconflicting features is necessary to have a good classification result.

### 2.4. Existing Natural Language Processing Platforms

Because of commercial opportunities, many technology giants have developed NLP platforms and opened them to the public. Examples include the IBM Watson [28,29], Facebook Wit.ai [30], Microsoft LUIS [31,32], Amazon Lex, Google Dialogflow, and so on. To have a quick development of an NLP application, developers tend to leverage the free NLP platforms. With the support of these platforms and associated tools, the process of development and data training becomes efficient. However, the major drawback of using these platforms is the limited tuning parameters. It could become a serious problem when there is a need for performance tuning. Therefore, sometimes developers need to pay to have additional flexibilities. In this work, the proposed method was compared with Facebook Wit.ai because it provides more flexibilities than other platforms in performance tuning.

## 3. Methodology

### 3.1. Design Requirements and Challenges

This work aims to design DBOS, a user-friendly and effective dialog-based object query system, for hospital nurses. In order to shorten the learning curve and therefore reduce the training cost, it is highly preferred to integrate the system with a mobile app that is used daily by nurses. For this, Line, a popular free app for instant communication, was selected in the implementation of DBOS. When given a search query, DBOS should correctly identify user intent and return the user with a proper result.

However, due to the nature of nurses’ busy work, the query input may be short or incomplete, which makes it challenging for traditional NLP methods to identify query intent. According to the discussion in the previous section, machine-learning-based methods could have difficulties in classifying a brief query intent because the shorter the sentence is, the fewer features there will be. Rule-based methods are also excluded since enumerating all possible rules is time consuming and impractical in the target application. Feature-based methods that treat each word as equally important could fail in intent classification. To overcome this challenge, DBOS combines CS and TF-IDF to correctly classify user intent. Detailed information is presented in the following subsections.

### 3.2. DBOS Overview

**Figure 1:**
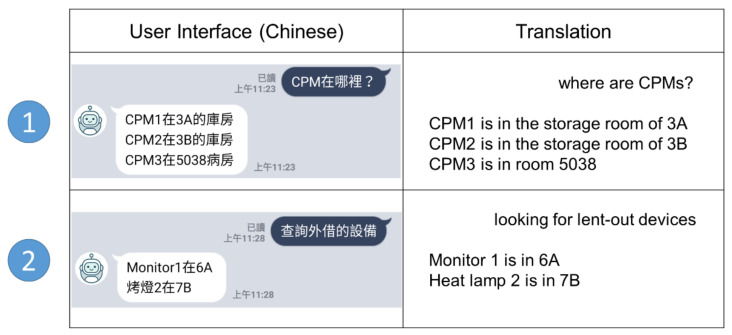
Dialog-based object query system (DBOS) user interface.

DBOS is a user-friendly object query system integrated with Line. The kernel of DBOS is a specially designed NLP algorithm that can determine a user’s intent and return the location of found objects. By enabling the voice-to-text function of a smartphone, DBOS can handle both Chinese text input and voice input. The voice-to-text feature is particularly convenient and efficient for nurses when they walk around rooms in a ward or multitask under pressure. Figure 1 shows two use cases of device searching through Line’s interface. The first one shows the search for a CPM (continuous passive motion) device while the second one shows the search for borrowed devices.

**Figure 2:**
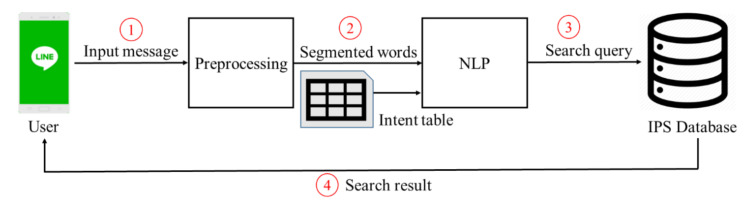
The architecture of DBOS.

Figure 2 is the architecture of the DBOS, which includes four steps. At the beginning, the preprocessing module takes the user’s Chinese string as input and makes it digestible for the following NLP (Step 1). In the implementation, Jieba [8] is used for sentence segmentation and redundant word removal. Based on the result from Jieba, the empty string, punctuation, stop words, and redundant words are removed. In addition, lowercase conversion is performed when there are English words in the input string, such as equipment name. The segmented words and the intent table are taken as input by NLP (Step 2), in which the intent table is a list of predefined user intent used for similarity comparison. The user intent with the highest matching score is sent to an IPS (indoor positioning system) database (Step 3). Finally, the search result is returned to the user through Line’s interface (Step 4). DBOS is an extension of our previous work, the SBOT [7], which combined the popular social media app Line with an indoor positioning system. In Figure 2, Steps 1, 2, and 3 are the new content in this work, while Step 4 is covered by the SBOT previously. The user interface, shown in Figure 1, was built on top of the SBOT. For readers who are interested in how the SBOT returns the search result, please refer to [7].

**Figure 3:**
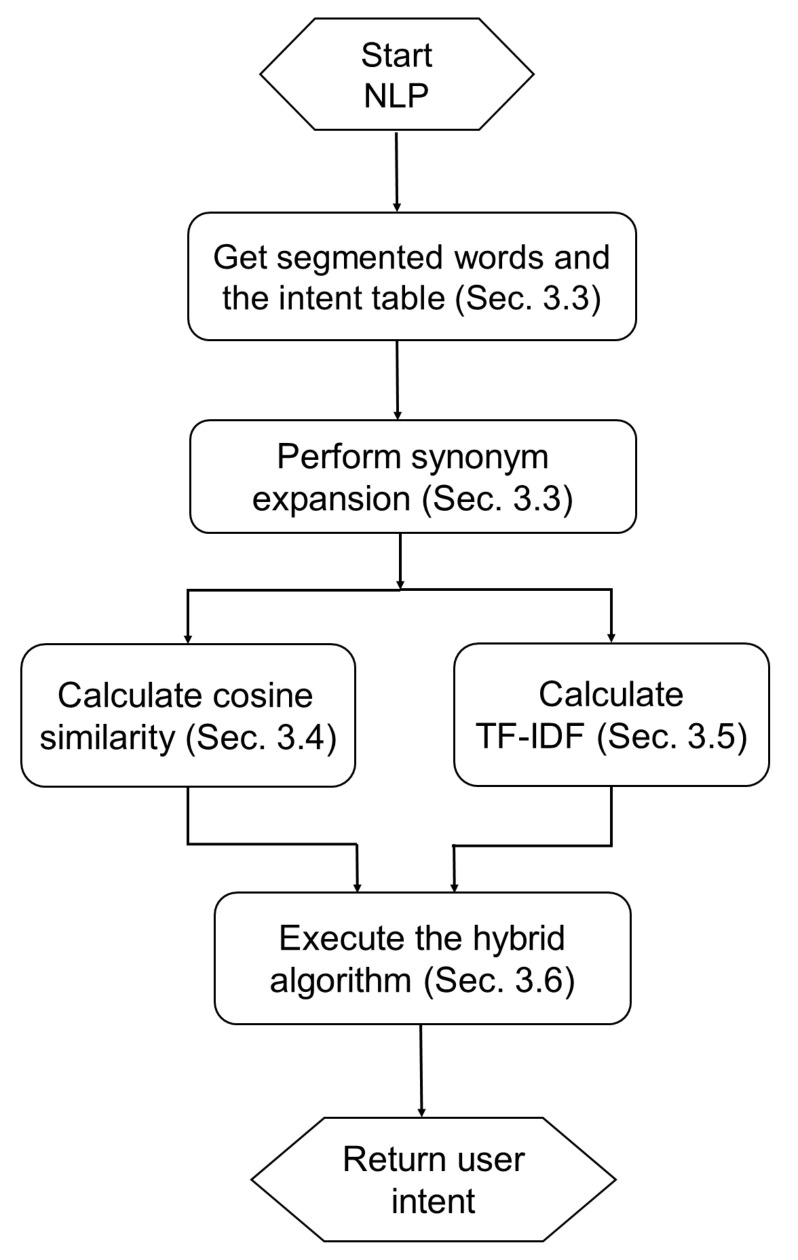
The flowchart of natural language processing (NLP).

Figure 3 shows the flow of NLP, in which NLP first takes segmented words and the intent table as input. Then, synonym expansion is performed before the calculation of cosine similarity and TF-IDF. Finally, based on the results of cosine similarity and TF-IDF, a hybrid method is executed to determine the user intent. The details of each step are described in the following subsections.

### 3.3. Intent Table and Synonym Expansion

The intent table contains the possible intents queried by nurses in their daily work. To ensure the completeness of the intent table, an experienced head nurse was consulted about how devices and equipment are managed in a hospital ward. Based on her feedback, users’ intents can be classified into three categories: finding devices, finding patients, and getting statistic reports. From this, 12 kinds of user intent were identified and are listed in Table 1. The intent table was used to compare with the user’s input sentence to determine the user’s intent.

Different users may use a different wording to describe the same thing. To avoid a possible incorrect judgement of user intent, DBOS performs synonym expansion before calculating the similarity score of the user’s sentence. In other words, for a word in the user’s sentence, if its synonym is included in the intent table, the word will be replaced by the synonym. In the implementation, Jieba’s library and specially-defined words are used for synonym expansion. For example, “庫房” (storeroom) is a synonym of “堆房” (stockroom). If the user’s input sentence is “堆房剩餘設備” (unused equipment in the stockroom), it will be replaced by “庫房剩餘設備” (unused equipment in the storeroom). Moreover, if there is an English word in the user input, such as CPM, it will be regarded as a piece of equipment.

### 3.4. Cosine Similarity

**Figure 4:**
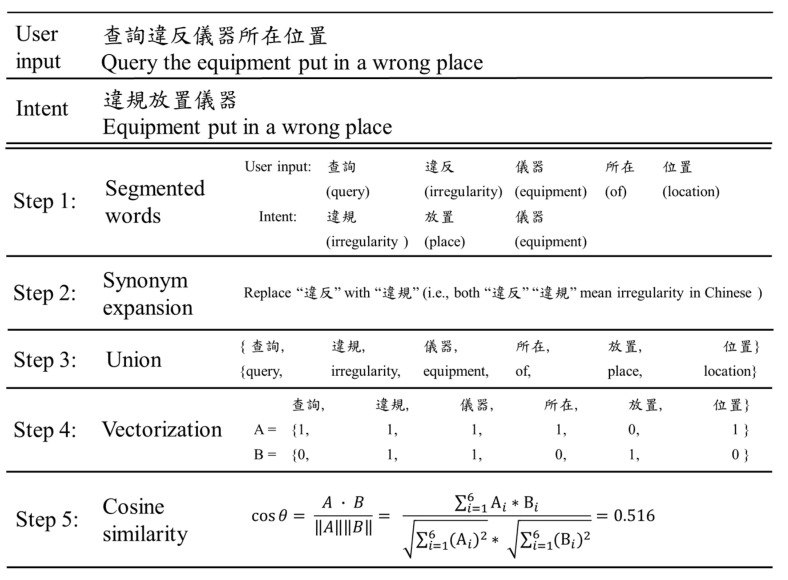
An example of cosine similarity.

Cosine similarity (CS) is a method in determining the similarity of two given strings. In the target application, the two strings are the user’s input and a kind of user intent in the intent table. The closer the value of cosine similarity is to 1, the more similar the two strings are. An example shown in Figure 4 illustrates how CS works. The user’s input is “查詢違反儀器所在位置” (query the equipment put in a wrong place), which is compared with intent No. 7 “違規放置儀器” (equipment put in a wrong place) of Table 1. First, both the user input and intent are segmented into words. Then, “違反” is replaced by “違規” in the step of synonym expansion (Step 2). Further, a union is formed to collect all segmented words of the two strings (Step 3). In the step of vectorization, the user input vector A and the intent vector B are developed depending on whether a word is in the union. The value of “1” indicates that the word is in the union while “0” indicates that the word is not in the union (Step 4). The cosine similarity is determined by the following:

(1) $$\cos\theta =\;\frac{A\;\cdot \;B}{\left\|A\|\|B\right\|}=\frac{\sum_{i=1}^{n}A_{i}*B_{i}}{\sqrt{\sum_{i=1}^{n}{\left(A_{i}\right)}^{2}}*\;\sqrt{\sum_{i=1}^{n}{\left(B_{i}\right)}^{2}}}\;$$

in which, *A* = {1, 1, 1, 1, 0, 1}, *B* = {0, 1, 1, 0, 1, 0}, and *n* is 6. The similarity score of the two vectors is 0.516. The same process repeats until the user string has been compared with each predefined user intent listed in the intent table. In this example, both the CS scores of intents No. 2 and No. 7 are 0.516. They are also the highest among all CS scores, which makes it difficult for CS to determine the user intent. The major reason is that CS treats each segmented word as equally important. To overcome this limitation, TF-IDF is considered in the next subsection.

### 3.5. TF-IDF

TF-IDF is another method in determining the similarity of the two given strings. Unlike CS, TF-IDF takes into account the term frequency, i.e., the number of times that a word occurs in the currently compared user intent. For TF-IDF, the lower the term frequency of a word is, the more unique (or important) the word will be. In the process of user intent identification, a word with a low term frequency is more important than one with a high term frequency.

Let $a_{i}$ denote the $i^{th}$ segmented word in the user input. The $T_{j}$ is the $j^{th}$ intent in the intent table. The total number of user intents is N, and the number of words in $T_{j}$ is $w_{j}$. In addition, $c_{i,j}$ is the count of $a_{i}$ which is the number of times that $a_{i}$ occurs in $T_{j}$. The $c_{i}^{’}$ is the intent number listed in the intent table that contains $a_{i}$. The term frequency (TF) of $a_{i}$, denoted by $f_{i,j}$, is determined by $c_{i,j}\;/\;w_{j}$. In addition, the IDF (inverse document frequency) of $a_{i}$, denoted by $d_{i,j}$ is determined by $\log\left(N/c_{i}^{’}\right)$. For TF-IDF, the rarer $a_{i}$ is, the higher $d_{i,j}$ is, and the more important $a_{i}$ is. Finally, the TF-IDF score of $a_{i}$ is calculated by $f_{i,j}*d_{i,j}$.

**Figure 5:**
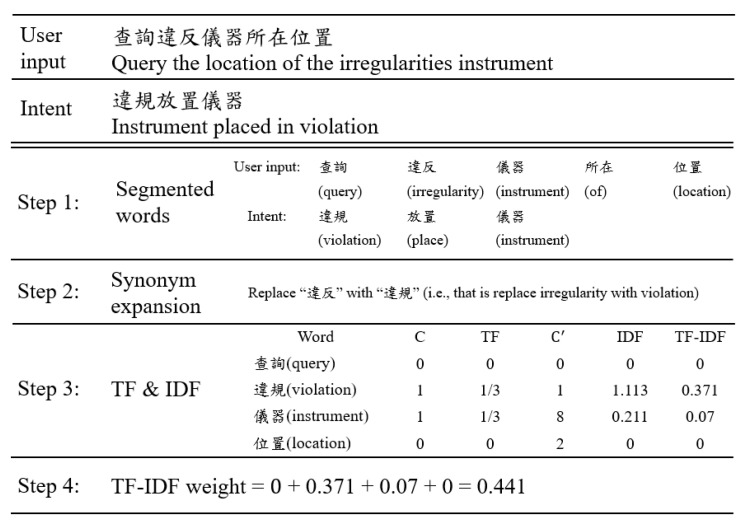
Example of term frequency–inverse document frequency (TF-IDF).

In Figure 5, the same user input is used as that in Figure 4 to illustrate how TF-IDF works. In this example, N is 12 since there are twelve kinds of user intents in Table 1. The phrase “違規放置儀器” (equipment put in a wrong place) is the 7th intent and $w_{7}\;$= 3. The first two steps of TF-IDF are the same as those of CS. It should be noted that in this work, “irregularity” and “violation” are synonyms in Chinese. In addition, “equipment” and “instrument” are synonyms in Chinese. If the word “irregularity” is in the user’s input, it will be replaced by “violation” in Step 2 (i.e., synonym expansion). Similarly, if the word “equipment” is in the user’s input, it will be replaced by “instrument”. In Step 3, the values of TF and IDF are calculated before the value of TF-IDF is determined. For example, “查詢” (query) is the first word that is denoted by $a_{1}$. Since $a_{1}$ does not occur in $T_{7}$, the count, TF, IDF, and TF-IDF are 0. For the second word $a_{2}$ “違規”, $c_{2,7}=1$, $f_{2,7}=c_{2,7}/w_{7}=1/3$, and $c_{2}^{’}=1$, $d_{2,7}=\;\log\left(N/c_{2}^{’}\right)=\log\left(13/1\right)\;=1.113$. Then, the TF-IDF weight of $a_{1}$ is 0.371 (= 0.333*1.113). As the last column of Step 3 shows, TF-IDF increases the weight of “違規” (irregularity) while it decreases the weight of “儀器” (equipment), even though the term frequencies of the two words are the same. In Step 4, all weights are added up, and the TF-IDF score is 0.441. The user’s input is compared with each user intent listed in Table 1. Finally, the intent with the highest TF-IDF score is selected. In this example, 0.441 (intent No. 7) is the highest among all TF-IDF scores, and TF-IDF successfully matches the user query to the correct user intent, i.e., No. 7. The major reason for this is that TF-IDF properly adjusts the weight of each word.

Although TF-IDF succeeds in the above example, it may fail if the importance of a word is not related to its term frequency. For example, for the user input “查詢儀器位置” (query for the location of equipment), TF-IDF will raise the weight of “位置” (location) rather than the “儀器” (equipment) and result in an inaccurate classification. On the contrary, CS can successfully identify the above query. Because of the uncertainly of CS and TF-IDF in classifying user intent, a hybrid method is proposed to address this problem in the next subsection.

### 3.6. Hybrid Method

In the hybrid method, when given a user query q, CS and TF-IDF are used to respectively measure the similarity between q and each user intent listed in the intent table. The number of user intents is N, and the *j*th user intent is $u_{j}$. Again, $a_{i}$ denotes the *i*th segmented word in q, and $c_{i}^{’}$ is the number of intents that contain $a_{i}$. If $c_{i}^{’}$ is 1, then $a_{i}$ is called a low-frequency word. The p is used to represent the percentage of the low-frequency words in q. Let $C_{j}$ denote the CS score of $u_{j}$ and $F_{j}$ the TF-IDF score of $u_{j}$, in which j is 1, 2, … , *N*. For ease of presentation, α is used to represent the index of the user intent with the highest scores among all CS cores, and α is determined by

(2) $$\alpha =\arg\underset{j=1,\;2,\;\ldots .,N}{\max}\left\{C_{j}\right\},$$

In addition, β is used to represent the index of user intent with the highest scores among all TF-IDF scores, and β is determined by

(3) $$\beta =\arg\underset{j=1,\;2,\;\ldots .,N}{\max}\left\{F_{j}\right\}$$

In other words, CS suggests $u_{\alpha }\;$as the user intent while TF-IDF suggests $u_{\beta }\;$as the user intent.

Let r represent the classification result of the user query. The hybrid method is shown in Algorithm 1, in which if α is equal to β or if $C_{\alpha }$ is larger than 2/3 (line 2), the classification result is $u_{\alpha }$, that is, $r=u_{\alpha }$. The former condition shows that both CS and TF-IDF suggest the same user intent while the later condition indicates that most parts of the user query are similar to $u_{\alpha }$. When either of the two conditions holds, the hybrid method returns $u_{\alpha }$. On the contrary, if neither of the two conditions holds, the hybrid method further considers the result $u_{\beta }$ recommended by TF-IDF. Since TF-IDF amplifies the weights of low-frequency words, taking the suggestion provided by TF-IDF as the result should be done carefully. If the percentage p of the low-frequency words in q is larger than 50% but the CS similarity is less than 50%, there is a high possibility that the user intent $u_{\beta }\;$suggested by TF-IDF is wrong. If that is the case, $u_{\alpha }$ is taken as the result (line 5). In the following, the hybrid method is used to re-examine the examples shown in Section 3.4 and Section 3.5.
**Algorithm 1** Hybrid methodInput: $\left\{C_{1},\;C_{2},\;C_{3},\;\ldots ,\;C_{N}\right\}$$,\;\left\{F_{1},\;F_{2},\;F_{3},\;\ldots ,\;F_{N}\right\}$Output: r1:Determine α and β by Equations (2) and (3);2:$\mathrm{if}\;(\alpha ==\;\beta \;or\;C_{\alpha }\geq 2/3)\;$3: $r=\;u_{\alpha }$;4:else if
$\left(p\geq 1/2\;and\;C_{\alpha }\leq \;1/2\right)$
5: 
$r=\;u_{\alpha };$
6:else7:
 $r=\;u_{\beta };$
8:return
r;


The first example is “查詢違反儀器位置” (query the location of the irregularities instrument), in which CS failed but TF-IDF succeeded in determining the user intent. For CS, because both $C_{2}$ and $C_{7}$ are 0.516, so α can be 2 or 7, and CS cannot correctly determine the user intent. For TF-IDF, it suggested that $u_{7}$ is the user intent because $F_{7}=0.441$ is the largest among all $F_{j}$, where j is 1, 2, 3, …, 13. Thus, β is 7. For the hybrid method, since α can be 2 or 7, so α≠ β. In addition, $C_{2}=C_{7}<2/3$. Therefore, the user intent suggested by CS was not taken by the hybrid method (line 2). The user intent suggested by TF-IDF was further investigated (line 4). Since the low-frequency word ratio p is 1/4 and $C_{2}=C_{7}\geq 0.5$, the hybrid method determined $u_{7}$ as user intent (line 7), which is correct.

The second example is “查詢儀器位置” (query instrument location), in which CS succeeded but TF-IDF failed. For CS, it suggested $u_{2}$ as the user intent because $C_{2}=2/3$. On the other hand, TF-IDF suggested $u_{7}$ as the user intent because $F_{7}=0.441$. For the hybrid method, it took CS’s suggestion because $C_{2}\geq 2/3$. In both examples, the hybrid method successfully determined the user intent. A further comparison of the hybrid method and existing works will be given in the next section.

## 4. Experiments

### 4.1. Experiment Setup

In order to evaluate the effectiveness and practicality of the DBOS, an experienced head nurse’s queries for 12 types of user intent were collected first. Based on her queries, an additional 58 user queries were generated manually by rephrasing the head nurse’s sentences. Therefore, there are 70 user queries covering all types of user intent listed in Table 1. In the following subsections, the DBOS is first compared with CS and TF-IDF in Section 4.2. Then, the DBOS is compared with Facebook Wit.ai [33], a well-known open NLP engine, in Section 4.3. With the 70 user queries, the accuracy is defined as the percentage of the queries that are successfully identified. In the experiment, the identification is called successful if the user query is mapped to one and only one correct user intent. In other words, if the user query is mapped to more than one user intent or an incorrect user intent, the identification fails. It should be noted that this work is an extension of our previous work SBOT [7], which combined the popular social media app Line with an indoor object tracking system. For readers who are interested in the way how the indoor positioning system returns the location of a specific object when a user asked for the object, please refer to [7].

### 4.2. Comparisons of CS, TF-IDF, and Hybrid Method

This experiment evaluates the accuracy of CS, TF-IDF, and the hybrid method. The 70 testing user queries cover all 12 types of user intent listed in Table 1. The user queries are listed online for ease of reference [34]. Table 2 shows the results in which the tuple is defined as <the number of success cases >/<the number of testing cases>. For example, for the first intent, 6 user queries were used in the experiment. Both CS and the hybrid method can successfully recognize these user inputs while TF-IDF can only recognize 2 of them. The average accuracy of CS is 70%, TF-IDF is 74.2%, and the hybrid method is 92.8%. The results show that the hybrid method can improve the accuracy of classification by around 20%. The major reason is that the hybrid method considers both the text overlay of the compared two strings and the importance of each word.

On the contrary, CS treats all words as equally important, and this may result in an incorrect classification, especially when different types of intent contain the same words. The more identical the words shared by the different types of intent are, the more difficult it will be for CS to differentiate them. For example, in Table 2, “location” and “equipment” are included by many types of intent. When the “location” or “equipment” is in user’s input, there is a high possibility that CS points to a wrong user intent. In the experiment, most failed cases of CS are those where the similarity scores of two different types of user intent are the same. For example, given the user input “外借設備” (lent-out equipment), CS maps it to user intent “全部儀器設備” (No.1) and “外借或未歸還” (No.4) because their CS scores are both 0.408. The results confirm that it is important to consider how unique an input word is in user intent. Compared with CS, TF-IDF delivers a better performance as the term frequency is taken into account. However, the improvement is not that significant and consistent because TF-IDF could fail if a low frequency word occurs in more than two user intents. For example, given the user input “儀器所在位置” (query for the location of equipment), TF-IDF regards “位置” (location) as a low-frequency word and raises its weight. However, “位置” (location) occurs in two types of user intents (i.e., No. 2 and No.8), which makes it difficult for TF-IDF to differentiate them, especially when the length of each intent is different. If this is the case, CS scores should be considered. This also explains why DBOS outperforms CS and TF-IDF. According to our experiment, DBOS may fail in classifying user intent if there are too many occurrences of undefined words, such as the name of a piece of medical equipment, in the user input. The problem can be solved by further enriching the synonym dictionary. To further investigate the effect of the weighting parameter $C_{\alpha }$ of Algorithm 1 on the accuracy of the hybrid method, $C_{\alpha }$ is first set to 1/2, 3/4, and 4/5 respectively. According to experiment results, the accuracy of DBOS becomes 81.4% ($C_{\alpha }$ = 1/2), 75.7% ($C_{\alpha }$ = 3/4), and 77.1% ($C_{\alpha }$ = 4/5). We also set parameter $C_{\alpha }$ to around 2/3 (=0.67) at a finer granular, namely 0.64, 0.65, 0.66, 0.68, and 0.69. According to our experiment, the accuracy of DBOS remains at 92.8% when $C_{\alpha }$ is set to 0.64, 0.65, 0.66, 0.68, or 0.69. Since these settings do not further improve the accuracy of DBOT, $C_{\alpha }$ is kept at 2/3 (=0.67) in the implementation.

### 4.3. Comparison of Wit.ai and the Hybrid Method

Wit.ai is a well-known and open natural language processing platform provided by Facebook [35]. Wit.ai adopted machine-learning-based and rule-based methods to classify user intent. Wit.ai allows developers to define their own types of user intent. For each type of user intent, developers can input different sentences as training data to express the intent. The developers also have the flexibility to configure the strategies of entity extraction. In Wit.ai, an entity is a meaningful piece of information of a given sentence. In the usage scenario, an entity could be a noun such as “儀器” (equipment), “病人” (patient), and so on. Entities can be further classified as traits, free text, or keywords. A trait is an entity that cannot be inferred from the keywords or specific phrases in the sentence. A trait can also be a label that affects the meaning of the whole sentence, such as the emotion of the sentence. Free texts are specific parts of the sentence that need to be extracted. Keywords are the most relevant words in the sentence. Wit.ai provides the function of synonym expansion for developers to expand the union of keywords.

In the experiment, to have a fair comparison, the intent table of Wit.ai is the same as that of DBOS. In addition, 150 sentences were used as training data for Wit.ai. For each type of user intent, there are 10 sentences in average. Before feeding the training data to Wit.ai, we ensured that all the 150 sentences can be correctly identified by DBOS. In other words, all these sentences are meaningful, and each of them is mapped to one and only one type of user intent. For ease of reference, the 150 sentences that were used to train the Wit.ai model are available online [36]. After the Wit.ai was trained, another 70 user queries were used as testing data. The 70 user queries are the same as those used in Section 4.2.

Table 3 shows the experiment results, in which the accuracy of Wit.ai is 72.8% while that of DBOS is 92.8%. Obviously, DBOS performed either equal to or better than Wit.ai in all types of user intent. In addition, Wit.ai achieved 100% accuracy in only three types of user intent (i.e., user intent No. 2, No.5, and No.8) among the thirteen types of user intent. Compared to Wit.ai, DBOS delivers 100% accuracy in eight types of user intent. The major reason for Wit.ai’s poor performance is that most sentences are short sentences. The shorter a sentence is, the fewer features it contains, and the more difficult it is for Wit.ai to classify it. The situation becomes worse for Wit.ai if the sentence expressions of different types of user intent are similar. This happened for user intents No.9, No.10, and No.11, as several words are shared among these user intents, such as “儀器” (equipment) and “使用” (usage). The results also show that Wit.ai has difficulties in identifying Chinese grammar. For example, Wit.ai cannot differentiate the following two sentences that have the same meaning in Chinese: “查詢儀器” (query equipment location) and “儀器查詢” (query equipment location). On the contrary, DBOS does not have this problem since it considers similarity and term frequency rather than the rules in a language when classifying a user input. The effect of the number of expressions on the accuracy was also investigated. Generally speaking, the more training data Wit.ai receives, the more accurate it will be. However, as Table 4 shows, when the number of expressions is larger than 150, the improvement of the accuracy becomes uncertain according to the experiment. The major reason is that the user inputs are short messages in the application, and the expressions of different types of user intent are similar. Further increasing the amount of training data does not help much in improving the accuracy. Sometimes it could cause an opposite effect and decrease the accuracy.

## 5. Conclusions

In this work, DBOS, a dialog-based object query system, was proposed to provide nurses with voice and text inquiry services. In order to flatten the learning curve and create a user-friendly interface, DBOS was integrated with Line. Unlike existing NLP methods, DBOS first uses CS to calculate the text similarity between the user input and user intent. Next, TF-IDF adjusts the weight of each word in the user input according its term frequency. Finally, a hybrid method that considers the consistency of the suggestions of CS and TF-IDF and the similarity is used to determine user intent. In the design, if both CS and TF-IDF suggest the same user intent as the result, the user intent is determined. On the other hand, if there is inconsistency between CS and TF-IDF, DBOS considers the percentage of the low-frequency words followed by the CS similarity scores. In the experiment, DBOS was compared not only with feature-based methods (i.e., CS and TF-IDF) but also a machine-learning-based method, Facebook Wit.ai. The results show that DBOS achieved a 92.8% accuracy in determining user intent and outperformed the existing methods compared. In the future, DBOS will be further extended to other usage scenarios in a hospital, such as outsourced workers management, visitor management, and equipment usage management. In addition, investigating machine-learning methods for performance improvement and finding the optimal solution of the hybrid method will be considered.

## Figures and Tables

**Table 1:** Intent table.

No.	Intent	No.	Intent
1	全部儀器設備 (The location of all equipment)	8	病人位置 (A patient’s location)
2	儀器設備位置 (The location of a piece of equipment)	9	儀器使用次數 (The number of times the equipment is used)
3	損壞或壞掉儀器 (Damaged or broken equipment)	10	儀器總使用時間 (The total usage time of the equipment)
4	外借或未歸還 (Lent-out or not returned equipment)	11	儀器平均使用時間 (The average usage time of the instrument for each use)
5	送修維修等待修理儀器 (Equipment sent or will be sent for repair)	12	護理人員交班記錄報表 (Nursing shift report)
6	庫房剩餘庫存 (Equipment in the storeroom)	13	皆無匹配 (Nonmatch)
7	違規放置儀器 (Equipment put in a wrong place)		

**Table 2:** The accuracy of different methods.

No.	Query Scenarios (User Intent)	CS	TF-IDF	Hybrid (DBOS)
1	The location of all equipment	6/6	2/6	6/6
2	The location of a piece of equipment	4/4	0/4	4/4
3	Damaged or broken equipment	2/3	3/3	3/3
4	Lent-out or not returned equipment	4/12	10/12	10/12
5	Equipment sent or will be sent for repair	3/4	3/4	4/4
6	Equipment in the storeroom	3/6	5/6	5/6
7	Equipment put in a wrong place	4/7	4/7	6/7
8	A patient’s location	7/7	7/7	7/7
9	The number of times the equipment is used	8/8	8/8	8/8
10	The total usage time of the equipment	1/4	3/4	3/4
11	The average usage time of the instrument for each use	2/4	2/4	4/4
12	Nursing shift report	5/5	5/5	5/5
Accuracy		70% (49/70)	74.2% (52/70)	92.8% (65/70)

**Table 3:** Comparison of DBOS and Wit.ai.

NO.	Query Scenarios (User Intent)	Hybrid (DBOS)	Wit.ai
1	The location of all equipment	6/6	5/6
2	The location of a piece of equipment	4/4	4/4
3	Damaged or broken equipment	3/3	2/3
4	Lent-out or not returned equipment	10/12	8/12
5	Equipment sent or will be sent for repair	4/4	4/4
6	Equipment in the storeroom	5/6	5/6
7	Equipment put in a wrong place	6/7	4/7
8	A patient’s location	7/7	7/7
9	The number of times the equipment is used	8/8	6/8
10	The total usage time of the equipment	3/4	2/4
11	The average usage time of the instrument for each use	4/4	1/4
12	Nursing shift report	5/5	3/5
Accuracy		92.8% (65/70)	72.8% (51/70)

**Table 4:** The effect of the number of expressions on the accuracy of Wit.ai.

Number of Expressions (Training Data)	20	80	120	150	170
Accuracy	44.2%	62.8%	71.4%	72.8%	64.3%
